# STDP and STDP variations with memristors for spiking neuromorphic learning systems

**DOI:** 10.3389/fnins.2013.00002

**Published:** 2013-02-18

**Authors:** T. Serrano-Gotarredona, T. Masquelier, T. Prodromakis, G. Indiveri, B. Linares-Barranco

**Affiliations:** ^1^Department of Analog and Mixed-Signal Design, Instituto de Microelectrónica de Sevilla, IMSE-CNM-CSICSevilla, Spain; ^2^Unit for Brain and Cognition, Department of Information and Communication Technologies, Universitat Pompeu FabraBarcelona, Spain; ^3^Laboratory of Neurobiology of Adaptive Processes, UMR 7102, CNRS - University Pierre and Marie CurieParis, France; ^4^Centre for Bio-inspired Technology, Institute of Biomedical Engineering, Imperial College London; ^5^Institute of Neuroinformatics, University of Zurich and ETH ZurichZurich, Switzerland

**Keywords:** memristor/cmos, artificial-learning-synapses, spike-timing-dependent-plasticity, spiking-neural-networks

## Abstract

In this paper we review several ways of realizing asynchronous Spike-Timing-Dependent-Plasticity (STDP) using memristors as synapses. Our focus is on how to use individual memristors to implement synaptic weight multiplications, in a way such that it is not necessary to (a) introduce global synchronization and (b) to separate memristor learning phases from memristor performing phases. In the approaches described, neurons fire spikes asynchronously when they wish and memristive synapses perform computation and learn at their own pace, as it happens in biological neural systems. We distinguish between two different memristor physics, depending on whether they respond to the original “moving wall” or to the “filament creation and annihilation” models. Independent of the memristor physics, we discuss two different types of STDP rules that can be implemented with memristors: either the pure timing-based rule that takes into account the arrival time of the spikes from the pre- and the post-synaptic neurons, or a hybrid rule that takes into account only the timing of pre-synaptic spikes and the membrane potential and other state variables of the post-synaptic neuron. We show how to implement these rules in cross-bar architectures that comprise massive arrays of memristors, and we discuss applications for artificial vision.

## 1. Introduction

For many years, the field of neuromorphic engineering has struggled to develop practical neuro-computing devices that mimicked the principles and operations of biological brains, by directly exploiting the physics of electronic devices in mixed analog/digital VLSI (Indiveri and Horiuchi, 2011). However, there always was a clamor for a compact and distributed non-volatile memory, possibly tightly coupled to the signal processing components (neurons), so that the biological synapses counterparts could be properly emulated. The recent advent of nanoscale memristive-like devices (Wuttig and Yamada, 2007; Strukov et al., 2008; Yang et al., 2008; Jo et al., 2010; Govoreanu et al., 2011; Lee et al., 2011; Chanthbouala et al., 2012; Kuzum et al., 2012; Prodromakis et al., 2012a) opens the possibility of large-scale bio-inspired neural network implementations with minimal size-requirements for those elements in the circuit that are most numerous and therefore most space-intense: plastic synaptic connections. The strength of a synaptic link between two neighboring neurons depends on its history and more explicitly by the overall amount of neurotransmitters that has been propagated through it after a relevant neural spike. In similar fashion, the strength of a **memristor**, i.e., its memristance (or instantaneous resistance) is dictated by the amount of charge *q* that has flown through it or the accumulated voltage flux ϕ. Additionally, the intrinsic non-linear nature of practical solid-state memristors resembles the behavior of neural synapses.

On the other hand, and independently of the new nanoscale devices availability, the neuromorphic engineering field evolved naturally toward circuits and systems exploiting spiking signal encoding, as in biology. For example, a large collection of spike-driven vision sensors have been reported, such as sensors for luminance (Culurciello et al., 2003; Chen et al., 2011), temporal contrast (Barbaro et al., 2002; Mallik et al., 2005; Chan et al., 2007a; Lichtsteiner et al., 2008; Leñero-Bardallo et al., 2011; Posch et al., 2011; Serrano-Gotarredona and Linares-Barranco, 2013), motion (Kramer, 1996; Sarpeshkar et al., 1996; Ozalevli and Higgins, 2005), and spatial contrast (Ruedi et al., 2003; Zaghloul and Boahen, 2004; Costas-Santos et al., 2007; Massari et al., 2008; Leñero-Bardallo et al., 2010). Spike-driven principles have also been used for auditory systems (Sarpeshkar et al., 2005; Wen and Boahen, 2006, 2009; Chan et al., 2007b), competition and Winner-Take-All networks (Indiveri, 2000; Chicca et al., 2007; Oster et al., 2008), learning (Mill et al., 2011), classification (Mitra et al., 2009), fall detection (Fu et al., 2008), and systems distributed over wireless sensor networks (Teixeira et al., 2005; Massari et al., 2008). Apart from real-time sensing, spike-driven processing systems can produce extremely fast responses. Examples of spike-driven processing modules (chips) are those that, emulating biological neocortical structures, perform spatio-temporal feature extraction such as fixed-kernel (Venier et al., 1997; Choi et al., 2005) or programmable kernel (Serrano-Gotarredona et al., 2006; Camuñas-Mesa et al., 2011, 2012) 2D convolutions, and generic massive neural processing (Vogelstein et al., 2007; Fieres et al., 2008; Khan et al., 2008; Serrano-Gotarredona et al., 2009; Zamarreno-Ramos et al., 2012).

Unavoidably, the learning capability is one key characteristic that is required for building cognitive artificial neural systems. Recently proposed artificial neural processing systems spend great resources for this task: the multi-million European initiative *FACETS/BrainScales* (Fieres et al., 2008) is developing a 200.000 neuron wafer^1^ where most of the silicon area is used for implementing **Spike-Timing-Dependent-Plasticity (STDP)** learning mechanisms in the synapses. The UK initiative *SpiNNaker* (Khan et al., 2008) based on multi-processors ARM technology has to use hybrid packaging technology in order to encapsulate two separate Silicon chips into each chip package: one chip is being the genuine SpiNNaker chip with 18 ARM^2^ CPUs, and the second chip being a commercial 128MB DRAM chip for local synaptic storage. Both the learning mechanisms and the storage of learned parameters require substantial silicon real-estate in traditional silicon-based chip technology.

However, the advent of new nanoscale technologies has shed new expectations, giving hopes for the development of ultra-compact, fast and efficient learning and storage mechanisms that may result in affordable, low power, compact, large scale, artificial neural systems (Wuttig and Yamada, 2007; Strukov et al., 2008; Yang et al., 2008; Jo et al., 2010; Govoreanu et al., 2011; Lee et al., 2011; Chanthbouala et al., 2012; Kuzum et al., 2012; Prodromakis et al., 2012a). A very promising new class of nanoscale devices is the one that comprises the so called *memristors* (Chua, 1971; Chua and Kang, 1976; Strukov et al., 2008; Borghetti et al., 2009; Jo et al., 2009, 2010), whose distinct characteristic is that they have ***mem**ory* while they operate like variable two-terminal *res**istors***. It was recently postulated that such tiny nanoscale devices, when driven by appropriately shaped voltage pulses, could be embedded within traditional CMOS^3^ microchips, resulting in truly asynchronous^4^ artificial learning neural “tissue” equipped with STDP (Linares-Barranco and Serrano-Gotarredona, 2009b,a; Zamarreño-Ramos et al., 2011).

Although this still needs to be proven experimentally and all practical limitations are yet to be identified, while memristors are continuously being improved and optimized over many labs worldwide, the potential of building very dense hybrid memristive-CMOS learning systems is there. The resulting implementations can be extremely compact STDP-equipped systems, which contrast with pure CMOS-based attempts that either have resulted in physical STDP synapses consuming significant chip real-estate (Fieres et al., 2008) or complex computational work arounds in more algorithmic solutions (Rast et al., 2010; Davies et al., 2012). In this paper we quickly review the basic principles behind exploiting memristance for asynchronous STDP and extend the original findings to other types of STDP. In the next section we quickly review the memristor concept as well as some of the postulated physical mechanisms responsible for its operation. After this, section 6 summarizes STDP and some variation of it, as well as additive, multiplicative and quadratic STDP. Sections 4 and 5 review how to combine memristors with specific CMOS neurons to result in different types of STDP. Section 6 mentions an application in the context of artificial vision, and section 7 provides conclusions.

## 2. Memristors

**Figure 1:**
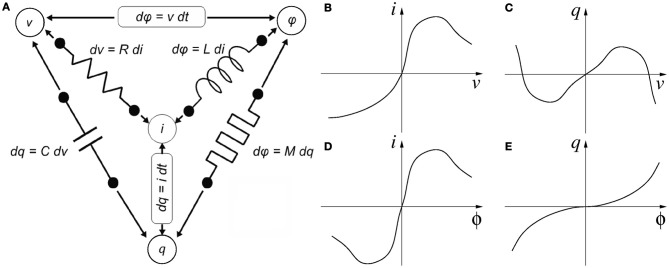
**Four variables of circuit theory linked by six mathematical relations consisting of the functional relationships of the four passive circuit elements, Faradays law of induction and the definition of electric current. (A)** Chua's symmetry argument and **(B–E)** descriptions of the four canonical two-terminal devices. **(B)** A resistor is defined by a static relationship between a device's voltage and current. **(C)** A capacitor is defined by a static relationship between a device's charge and voltage. **(D)** An inductor is defined by a static relationship between a device's current and flux. **(E)** And a memristor is defined by a static relationship between a device's charge and flux.

Memristance was postulated in 1971 by Chua (1971) as the fourth missing canonical circuit element through his famous symmetry argument, illustrated in Figure 1. According to circuit theoretical fundamentals, there are four basic electrical quantities (Chua et al., 1987): (1) voltage difference between two terminals “*v*,” (2) current flowing through into a device terminal “*i*,” (3) charge flowing through a device terminal or integral of current *q* = *∫i*(τ)*d*τ, and (4) flux or integral of voltage ϕ = *∫v*(τ)*d*τ. A two-terminal device is said to be canonical (Chua et al., 1987) if either two of the four basic electrical quantities are related by a static^5^ relationship, as shown in Figure 1. A resistor has a static relationship between terminal voltage *v* and device current *i*, as shown in Figure 1B. A capacitor shows a static relationship between charge *q* and voltage *v*, as shown in Figure 1C. An inductor has a static relationship between its current *i* and flux ϕ, as shown in Figure 1D. These three devices have been very well known since the origins of Electronics and Electricity. However, there are other possibilities for combining the four basic electrical quantities: (*q*, *i*), (*v*, ϕ), and (*q*, ϕ). Ignoring the combinations of a quantity with its own time derivative leaves us with one single additional possibility: (*q*, ϕ). This reasoning led Chua to postulate the existence of a fourth basic two-terminal element, which he called the *Memristor*. *Memristors* behave as resistances in which the resistance changes through some of the basic electrical quantities, and is somehow *memorized*. The memristor would show a static relationship between charge *q* and flux ϕ, as shown in Figure 1E. If the *q* vs. ϕ relationship is linear, the *memristor* degenerates into a linear resistor. Although none of the so-far reported memristors can be described by a static constitutive relationship in the (*q*, ϕ) plane (and thus, strictly speaking, the 1971 fourth canonical element is still missing), they all fall within Chua's 1976 generalization of *Memristive Systems* (Chua and Kang, 1976). From here on we will use the term *memristor* for Chua's 1976 definition of *memristive system*. Consequently, the simple concept of *memristance* as defined in Figure 1D can be extended to refer to any device exhibiting resistive behavior (its *i*/*v* curves cross the origin) whose resistance can change through some of the four basic electrical quantities (or a combination of them, or their time derivatives or integrals, etc.), while at the same time exhibiting *memory* for that resistance. In that case, more elaborate mathematical descriptions are required (Chua and Kang, 1976).

**Figure 2:**
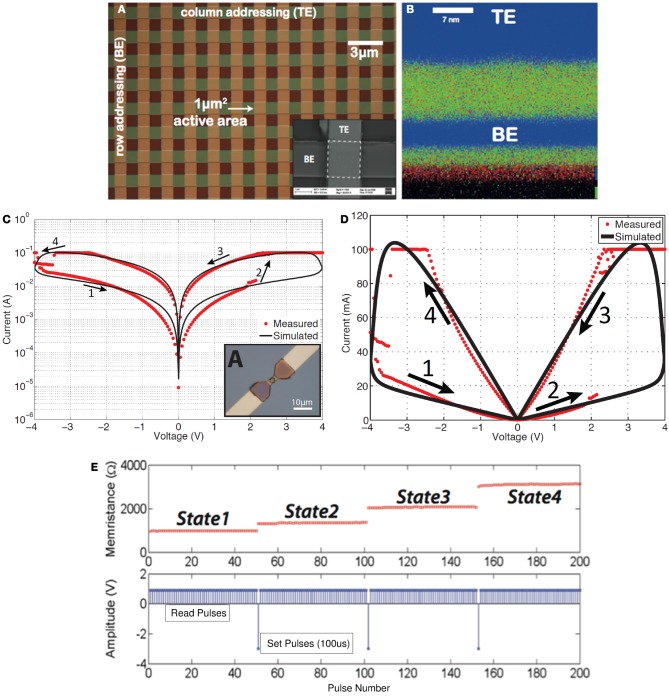
**Solid-state *TiO*_2_-based memristors fabricated at Imperial College London. (A)** Microphotograph of a memristor cross-bar array, with a close-up SEM illustration of a single cell appearing in the inset of **(A)**. **(B)** CHEMI-STEM map of a lamella cross-section of one of the devices shown in **(A)**: blue denotes Pt (top and bottom electrodes) while green and red correspond to *T*i and *O*_2_ species (Prodromakis et al., 2012a). **(C)** Simulated and measured pinched hysteresis I-V characteristics (absolute memristor current |I| vs. signed memristor voltage V) (Prodromakis et al., 2011) in log scale or **(D)** linear scale, and **(E)** multi-state programming of a *TiO*_2_-based memristor: read pulses are positive and small amplitude (1V) that do not alter the resistance (memristance) of the memristor, while successive set pulses have negative high amplitude (3V) and do progressively alter the resistance (memristance) of the memristor.

Memristance has recently been demonstrated (with extraordinary impact among the research community) in nanoscale two-terminal devices, such as certain titanium-dioxide (Strukov et al., 2008; Borghetti et al., 2009; Prodromakis et al., 2011, 2012a) and amorphous Silicon (Jo et al., 2009) cross-point switches. However, memristive devices were reported earlier by other groups (Argall, 1968; Prodromakis et al., 2012b). Memristance arises naturally in nanoscale devices because small voltages can yield enormous electric fields that produce the motion of charged atomic or molecular species, changing structural properties of a device (such as its doping profile) while it operates. Its functional characteristic has been a pinched hysteresis loop in the i–v domain (Figures 2C,D); a signature that has been observed in various dissipative devices (Prodromakis et al., 2012b). Particularly nowadays various emerging resistive random-access memory (ReRAM) nano-devices (Chua, 2011), with one scaling extreme being the atomic switch (Terabe et al., 2005), are classified as being memristors, and show attributes that resemble biological synapses (Ohno, 2011) providing exciting prospects for demonstrating neuromorphic applications (Avizienis et al., 2012). Hysteresis is typically noticed in systems/devices that possess certain inertia, causing the value of a physical property to lag behind changes in the mechanism causing it; manifesting memory (Pershin and Di Ventra, 2011). Particularly in the case of nanoscale memristors, this inertia has been ascribed to Joule heating (Fursina et al., 2009), the electrochemical migration of oxygen ions (Nian et al., 2007) and vacancies (Yang et al., 2008), the lowering of Schottky barrier heights by trapped charge carriers at interfacial states (Hur et al., 2010), the phase-change (Wuttig and Yamada, 2007), the formation/rupture of conductive filaments (Kwon et al., 2010), Yang et al. (2012) in a device's core, or even to some extent a combination of the aforementioned switching mechanisms.

Clearly, the impact of memristors is foreseen to be realized through their nanometric dimensions (see Figure 2B which is a cross section of one of the structures in Figure 2A), their capacity to store multiple bits or a continuum of information per element (Figure 2E) and the minuscule energy required to write distinct states, resulting in high spatial- and high storage-density well beyond the current state-of-the-art (Govoreanu et al., 2011). Nonetheless, the fact that the functional properties of such elements are associated with rate-limiting (frequency-dependent) electro- or thermo-dynamic changes that are contingent on both the present as well as the past environment, presents us with opportunities in exploiting them as novel computation elements.

**Figure 3:**
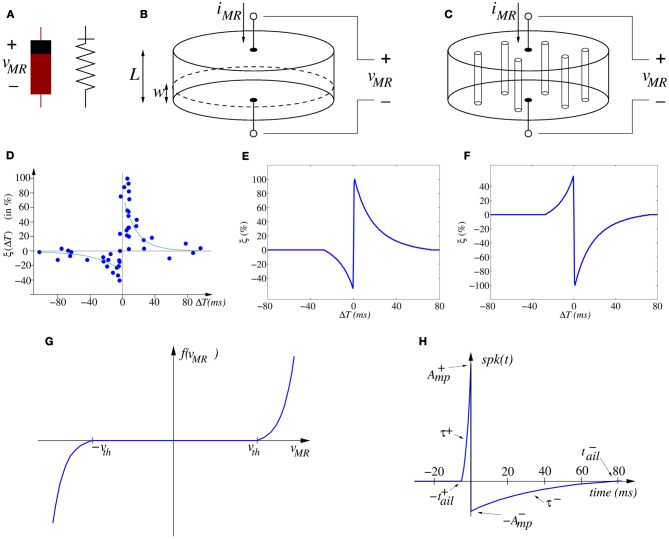
**(A)** Memristor asymmetric symbols. **(B)** Illustration of moving wall model describing memristor operation as two variable resistors in series. **(C)** Illustration of filament formation/annihilation model describing memristor operation as two variable resistances in parallel. **(D)** Experimentally measured STDP function ξ (Δ*T*) on biological synapses (data from Bi and Poo, 1998, 2001). **(E)** Ideal STDP update function used in computational models of STDP synaptic learning. **(F)** Anti-STDP learning function for inhibitory STDP synapses. **(G)** Shape of memristor weight update function *f*(v_MR_), **(H)** spike-shape waveform.

By definition, memristors can be either voltage/flux driven or current/charge driven. Depending on the polarity of the set and reset potentials required to change resistive states (RS), the devices can be classified as unipolar (URS) or bipolar (BRS) (Schindler et al., 2007) and consequently, their circuit symbol must indicate somehow their polarity, as depicted in Figure 3A. Voltage/flux driven memristors can be described by (Chua and Kang, 1976)

(1) $$i_{\text{MR}}=G(w,\;v_{\text{MR}},\;t)v_{\text{MR}}$$

(2) $$\dot{w}=f(w,\;v_{\text{MR}},\;t)$$

while current/charge driven memristors would be described as (Chua and Kang, 1976)

(3) $$v_{\text{MR}}=R(w,\;i_{\text{MR}},\;t)i_{\text{MR}}$$

(4) $$\dot{w}=f(w,\;i_{\text{MR}},\;t)$$

Here *w* represents some *structural* property parameter of the memristor. For example, in the 2008 HP paper (Strukov et al., 2008) the operation of the reported memristor was postulated as described by the *moving wall model* depicted in Figure 3B. In this simplified model a memristor of height *L*, sandwiched between two electrodes, has a low resistance region of height *w* and a high resistance region of height *L* − *w*. The memristor is considered to be divided into two regions. Both regions are separated by a boundary wall at position *w*, which moves up and down with the amount of charge that has flown through the memristor (in the case of being current/charge driven) or the accumulated flux (in case of being voltage/flux driven). The memristor would behave as two variable resistors in series. The total effective resistance of the memristor would be described by

(5) $$R=R_{\text{ON}}\frac{w}{L}+R_{\text{OFF}}\left(1-\frac{w}{L}\right)$$

This *moving wall model* can approximate phenomena like migration of oxygen ions (Nian et al., 2007) and vacancies (Yang et al., 2008), the lowering of Schottky barrier heights by trapped charge carriers at interfacial states (Hur et al., 2010), and the phase-change in some PCM (phase change materials) devices (Wuttig and Yamada, 2007).

However, resistive switching effects in dielectric-based devices have normally been assumed to be caused by conducting filament formation across the electrodes, although the understanding and modeling of these phenomena remains controversial. As a matter of fact, some researchers are observing the formation and annihilation of nanoscale width conducting filaments in memristors (Kwon et al., 2010; Yang et al., 2012). Precise modeling of this phenomenon is still under research (Shihong et al., 2012). However, let us here propose the following very simplified view to approximate this physical mechanism. Figure 3C illustrates schematically a memristor with several conducting filaments between the two electrodes. The number of filaments or their cross-sectional area would increase or decrease with memristor operation. Let us call now *w* the total cross sectional area of the effective conducting filaments at a given instant in time, and *S* the total cross section area of the memristor. The filaments present high conductivity (low resistivity), while the bulk presents much lower conductivity (high resistivity). All formed parallel filaments behave as one effective resistance of low resistance, while the rest of the bulk behaves as another higher resistivity resistor. Therefore, now the memristor behaves as two variable resistors in parallel. Consequently, its total conductance (inverse of resistance) could be described as

(6) $$G=G_{\text{ON}}\frac{w}{S}+G_{\text{OFF}}\left(1-\frac{w}{S}\right)$$

where *G*_ON_ is the conductance per effective cross section area of the filaments, and *G*_OFF_ is the conductance per effective cross section area of the filament-less bulk material. Parameter *w* would change from 0 to *w*_max_, the maximum possible effective cross section area of total conducting filaments (*w*_max_ ≤ *S*).

This changing cross section description not only approximates filament formation/annihilation phenomena, but also some other gradual cross section area variations observed in some phase-change or ferroelectric-domains-based materials (Chanthbouala et al., 2012).

As we will highlight later in sections 4 and 5, whether a memristor is better described by the moving wall model or the filament formation/annihilation model, impacts severely on the resulting type of STDP learning mechanism. The latter yields an additive type of STDP, while the former results in a quadratic type STDP. Note that a memristor can be either voltage/flux or current/charge driven, independently of whether it is a “wall” or a “filament” memristor.

## 3. Spike-timing-dependent-plasticity

STDP is the ability of natural or **artificial synapses** to change their strength according to the precise timing of individual pre- and/or post-synaptic spikes (Gerstner et al., 1993, 1996; Markram et al., 1997; Bi and Poo, 1998, 2001; Zhang et al., 1998; Feldman, 2000; Mu and Poo, 2006; Cassenaer and Laurent, 2007; Jacob et al., 2007; Young, 2007; Finelli et al., 2008; Masquelier et al., 2008, 2009). A nice overview of STDP and its history can be found elsewhere (Sjöström and Gerstner, 2010). STDP learning in biology is inherently asynchronous and on-line, meaning that synaptic incremental update occurs while neurons and synapses transmit spikes and perform computations. This contrasts to more traditional learning rules, like backpropagation (Rojas, 1996), where first neurons and synapses perform signal aggregation and neural state update (we call this here “performing phase”) and then synaptic updates are computed and applied (we call this here “weight update phase”) alternating these two phases during training. Even early proposals for memristor-based STDP learning implementations used artificial time-multiplexing to alternate continuously and synchronously between “performing” and “weight update” phases (Snider, 2008), thus requiring global system-wide synchronization. This can become a severe handicap when scaling up systems to arbitrary size. Here we show a fully asynchronous implementation for memristor-based STDP where “performing” and “weight update” phases happen simultaneously in a natural manner, as in biology (Linares-Barranco and Serrano-Gotarredona, 2009b,a; Zamarreño-Ramos et al., 2011), where there is no need for any global synchronization. Other researchers have proposed variations around these ideas (Bichler et al., 2012a; Kuzum et al., 2012).

Figure 3D shows the change of synaptic strength (in percent) measured experimentally from biological synapses as function of relative timing Δ*T* = *t*_pos_ − *t*_pre_ between the arrival time *t*_pre_ of a pre-synaptic spike and the time *t*_pos_ of generation of a post-synaptic spike. Although the data shows stochasticity, we can infer an underlying interpolated function ξ(Δ*T*) as shown in Figure 3E.

(7) $$\xi (\Delta T)=\{\begin{array}{cc} a^{+}e^{-\Delta T/\tau^{+}} & \text{if}\;\Delta T>0\\ -a^{-}e^{\Delta T/\tau^{-}} & \text{if}\;\Delta T<0 \end{array}$$

For a causal pre to post spike timing relation (Δ*T* > 0) the strength of the synapse is increased, while for an anti-causal relation (Δ*T* < 0) it is decreased. In the case of synapses with negative synaptic strength (as in some artificial realizations), the reversed version shown in Figure 3F can be used. Microchip CMOS circuit implementations of **STDP** rules that follow the description of Equation (7) have been reported (Indiveri et al., 2006), which result in about 30 transistors per plastic synapse, thus demonstrating the very high cost of their hardware realization. Let us call this *double-spike STDP*, since the weight will be updated after the arrival of the second spike (either pre- or post-synaptic).

Alternative variations of STDP have been proposed that do not require the intervention of both pre- and post-synaptic spikes (Brader et al., 2007), resulting in slightly less complex circuit implementations (Mitra et al., 2009). Let us call this *single-spike STDP*, since the weight will be updated after the arrival of pre-synaptic spikes only. This single-spike STDP rule updates the synaptic weight depending on the value of two local neural soma state variables. The first one is the membrane voltage *V*(*t*) and the second one is an auxiliary state variable *C*(*t*) proportional to the neuron's firing rate and equivalent to the biological neuron's Calcium concentration, which has the following dynamics

(8) $$\dot{C}=-\frac{C(t)}{\tau_{C}}+J_{C}\sum_{i}\delta (t-t_{i})$$

where *J*_*C*_ represents the contribution of one single post-synaptic spike and the time constant τ_*C*_ is comparable to the STDP learning window Δ*T*. The synaptic weight variable ξ is updated only when a pre-synaptic spike occurs at time *t*_pre_. The synaptic strength is increased or decreased by fixed size steps |*a*^±^| depending on the instantaneous values of *V*(*t*_pre_) and *C*(*t*_pre_) with respect to a given set of global thresholds {θ_*v*_, θ^*l*^_up_, θ^*h*^_up_, θ^*l*^_down_, θ^*h*^_down_}, as:

(9) $$\text{​​}\xi (t_{\text{pre}})=\{\begin{array}{c} a^{+}\text{ if }V(t_{\text{pre}})>\theta_{v}\text{ and}\\ \theta_{\text{up}}^{l}<C(t_{\text{pre}})<\theta_{\text{up}}^{h}\\ -a^{-}\text{ if }V(t_{\text{pre}})<\theta_{v}\text{ and}\\ \;\theta_{\text{down}}^{l}<C(t_{\text{pre}})<\theta_{\text{down}}^{h} \end{array}$$

Additionally, in this model the synaptic strength drifts slowly toward its upper or lower bound depending on whether it is above or below an intermediate threshold.

Both types of STDP rules, double-spike and single-spike, are very expensive to implement in conventional CMOS microchips (Indiveri et al., 2006; Fieres et al., 2008; Khan et al., 2008; Mitra et al., 2009). However, as we will see in the next section, both can be implemented with just one memristor per synapse if appropriate peripheral signal conditioning neurons are used in **hybrid CMOS/memristor** realizations.

Independently on whether STDP is either double-spike or single-spike, it is said to be either additive, multiplicative or quadratic if it additionally depends or not on the actual synaptic strength. If the STDP update is independent of the actual synaptic strength, it is said to be additive. Additive STDP requires the weight values to be bounded to an interval because weights will stabilize at one of their boundary values (van Rossum et al., 2000; Rubin et al., 2001). If the synaptic update is proportional to actual synaptic strength, it is called *multiplicative STDP* and weights may stabilize to values intermediate to their boundaries (van Rossum et al., 2000; Rubin et al., 2001; Gütig et al., 2003). If the synaptic weight update is proportional to the square of actual synaptic strength, we call it *quadratic STDP* (Zamarreño-Ramos et al., 2011).

## 4. Memristors and CMOS neurons for double-spike STDP

The more traditional double-spike STDP learning rule [as modeled by Equation (7)] can, in theory, be implemented by (Zamarreño-Ramos et al., 2011) (a) using a particular type of voltage/flux driven memristor (Jo et al., 2010) whose operation might be approximated by Equation (2) with (see Figure 3G)

(10) $$f(v_{\text{MR}})=\left\{ \begin{array}{l} I_{o}\text{ sign}(v_{\text{MR}})\left[e^{|v_{\text{MR}}|/v_{o}}-e^{v_{\text{th}}/v_{o}}\right]\\ \text{ if}|v_{\text{MR}}|>v_{\text{th}}\\ 0\text{ otherwise} \end{array}\right.$$

and bounded synaptic strength *w* ∈ [*w*_min_, *w*_max_], while (b) providing appropriately shaped pre- and post-synaptic spikes available at both synapse (memristor) electrodes (Zamarreño-Ramos et al., 2011). For example, consider a pair of identical pre- and post-synaptic spikes with a shape resembling that of biological spikes, with an on-set duration |*t*^+^_ail_| and a tail of duration |*t*^−^_ail_|, as shown in Figure 3H,

(11) $$\text{spk}(t)=\left\{ \begin{array}{l} \begin{array}{cc} \begin{array}{l} A_{\text{mp}}^{+}\frac{e^{t/\tau^{+}}-e^{-t_{\text{ail}}^{+}/\tau^{+}}}{1-e^{-t_{\text{ail}}^{+}/\tau^{+}}}\\ -A_{\text{mp}}^{-}\frac{e^{-t/\tau^{-}}-e^{-t_{\text{ail}}^{-}/\tau^{-}}}{1-e^{-t_{\text{ail}}^{-}/\tau^{-}}} \end{array} & \begin{array}{l} \text{if }-t_{\text{ail}}^{+}<t<0\\ \text{if }0<t<t_{\text{ail}}^{-} \end{array} \end{array}\\ 0\text{ otherwise } \end{array}\right.$$

**Figure 4:**
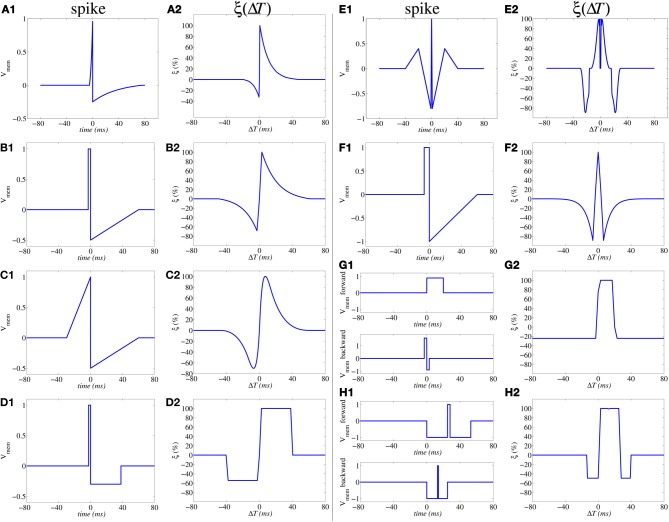
**Illustration of influence of action potential shapes on the resulting STDP memristor weight update function ξ(Δ *T*).** Memristor upper and lower thresholds are normalized to amplitudes ±1.0. From **(A1,A2)** to **(E1,E2)** the same spike waveform travels forward and backward. In **(F1,F2)** the forward and backward waveforms are the same but have opposite polarity. In **(G1,G2)** to **(H1,H2)** the forward and backward waveforms are different. In **(G1,G2)**, the positive pulse of the backward waveform exceeds amplitude +1.0, thus producing negative STDP update whenever there is a post-synaptic spike alone **(G2)**; otherwise if pre- and post-synaptic spikes happen within a given time window, there will be positive STDP update.

Under these circumstances, memristor voltage is *v*_MR_(*t*, Δ*t*) = α_pos_*spk*(*t*) − α_pre_*spk*(*t* + Δ*t*) and from Equations (2, 10) synaptic strength update can be computed as

(12) $$\text{​​}\Delta w(\Delta T)=\int f\left(v_{\text{MR}}(t,\;\Delta T)\right)dt=\xi (\Delta T)$$

which has been shown to result in the same shape illustrated in Figure 3E (Zamarreño-Ramos et al., 2011). Furthermore, by reshaping the spike waveform one can fine tune or completely alter the STDP learning function ξ(Δ t), as illustrated in Figure 4 (Linares-Barranco and Serrano-Gotarredona, 2009a). This way, by building neurons with a given degree of shape programmability, it is possible to change the STDP learning function at will, depending on the application, or make it evolve in time as learning progresses.

**Figure 5:**
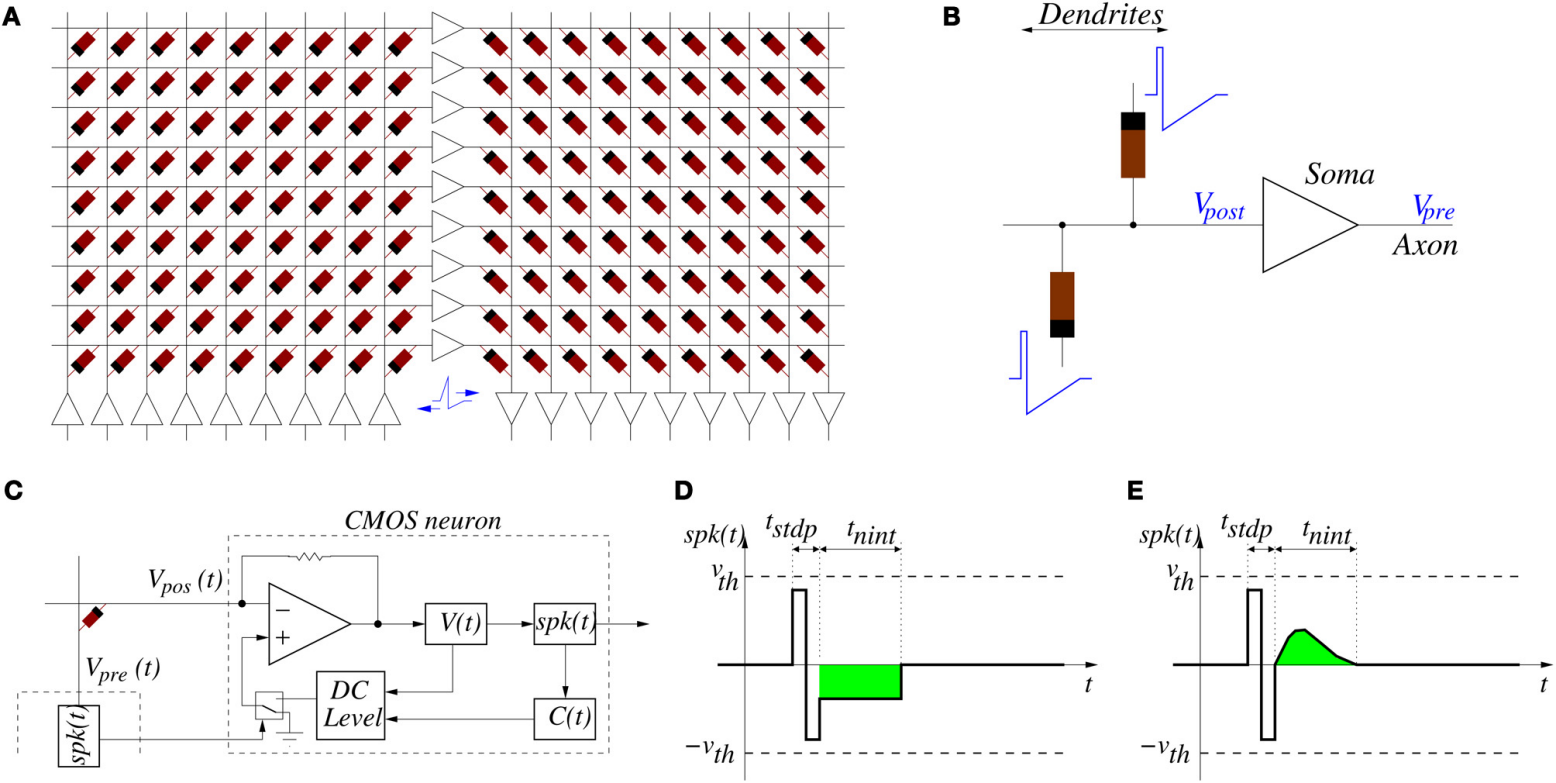
**(A)** Example of memristors and CMOS neuron circuits arrangement for achieving STDP learning: feed-forward neural system with three layers of neurons and two fully connecting synapse crossbars. **(B)** Details of parts around one post-synaptic neuron. While a neuron is silent, it sets a constant DC voltage at its input (*V*_post_) and output (*V*_pre_) nodes. When a neuron is sending a spike, it sets a voltage spike at both nodes. **(C)** Implementation of single-spike STDP: block diagram of CMOS neuron together with single memristor synapse connected between pre- and post-synaptic neurons, **(D)** example spike waveform with negative square neural activation shape, and **(E)** example spike waveform with positive more biological neural activation shape.

Figure 5A shows a way of interconnecting memristors and CMOS neurons for STDP learning. Triangles represent the neuron soma, being the flat side its input (dendrites) and the sharp side the output (axon). Dark rectangles are memristors, representing each one synaptic junction. Each neuron controls the voltage at its input (*V*_post_ in Figure 5B) and output (*V*_pre_ in Figure 5B) nodes. When the neuron is not spiking it forces a constant voltage at both nodes, while collecting through its input node the sum of input synaptic spike currents coming from the memristors, which contribute to changing the neuron internal state. When the neuron spikes, it sets a one-spike waveform at both input and output nodes. This way, they send their output spikes forward as pre-synaptic spikes for the destination synaptic memristors, but also backward to preceding synaptic memristors as post-synaptic spikes. Zamarreño et al. showed extensive simulations on these concepts, and how one can change from STDP to anti-STDP by switching polarities of spikes or memristors (Zamarreño-Ramos et al., 2011). For example, Figures 4F1,F2 illustrate the case where forward and backward spikes have opposite polarities, resulting in a symmetric STDP update function ξ(Δ*T*). Figures 4G1,G2 illustrate an example where forward and backward spikes are different, with the backward spike such that its positive part exceeds the positive memristor threshold (*v*_th_ = 1.0). This produces LTD (long term depression) or negative STDP update whenever there is a post-synaptic spike sufficiently apart from a pre-synaptic one; and produces LTP (long term potentiation) if pre- and post-synaptic spikes happen within a given time window (Bichler et al., 2012b,a). Figures 4H1,H2 illustrate a similar STDP update behavior, except that update (whether positive or negative) is restricted to a constraint time window.

If the system is structured into neural layers (for example, Figure 5A shows a 3-neuron-layer system) with **memristive synapses** in between, then for each layer all pre-synaptic neurons should have the same forward spike shape and all post-synaptic neurons should have the same backward shape. This way, all memristive synapses between these two neural layers will have the same STDP function ξ(Δ*T*).

### 4.1. Additive or quadratic STDP with memristors

In all these circuits, synaptic strength is the conductance *G* of the memristor: the higher the conductance of a memristor *G* is (or the lower its resistance *R* = 1/*G* is) the stronger the synaptic efficiency will be, as it will let more current through and thus affect more strongly the destination neuron state. Therefore, if the memristors used obey a “*moving wall*” model (see Equation 5), then STDP update Δ *w* = ξ(Δ*T*) changes wall position *w*, which from Equation (5) is directly proportional to resistance

(13) $$\begin{array}{l} \Delta R(\Delta T)=(R_{\text{ON}}-R_{\text{OFF}})\frac{\Delta w(\Delta T)}{L}\\ \;=\rho \xi (\Delta T) \end{array}$$

where ρ is a constant. Consequently, synaptic strength *G* = 1/*R* will change as

(14) $$\begin{array}{l} \Delta G(\Delta T)=-\frac{\Delta R(\Delta T)}{R^{2}}\\ \;=-G^{2}\Delta R(\Delta T)\propto -G^{2}\rho \xi (\Delta T) \end{array}$$

This means that synaptic strength update would follow a quadratic STDP learning rule.

If the memristor physics is better represented by the inter-electrode filament formation/annihilation model, then synaptic update would change parameter *w* of Equation (6), which is now directly proportional to memristor conductance (synaptic strength),

(15) $$\Delta G(\Delta T)=\frac{G_{\text{ON}}}{S}\Delta w(\Delta T)=\gamma \xi (\Delta T)$$

where γ is a constant. Therefore, synaptic update would be independent of actual weight (conductance) and the resulting STDP update rule is said to be of additive type. Note that Equations (10–12) and the resulting functions ξ(Δ*T*) in Figure 4 are common for both “wall” and “filament” models.

## 5. Memristors and CMOS neurons for single-spike STDP

For the case of the alternative single-spike STDP rule [as defined by Equations (8, 9)] we can use the same circuit topology shown in Figures 5A,B, but with different neuron circuits and spike shapes. Figure 5C shows one memristor connected between a pre-synaptic neuron generating voltage *V*_pre_(*t*) = *spk*(*t*) and a post-synaptic neuron that sets a given DC level at *V*_pos_(*t*). Figures 5D,E show possible waveforms for the output spike *spk*(*t*). It must be such that its amplitude is confined below the thresholds ±*v*_th_ of Figure 3G. We distinguish two parts: two sequential opposite sign square pulses during a time *t*_stdp_, and one synapse activation waveform for neural integration of duration *t*_nint_, with *t*_stdp_ « *t*_nint_. During times *t*_stdp_ the synapses connected to *V*_pre_ may experience weight update. During times *t*_nint_ the post-synaptic neurons would add the contribution of this pre-synaptic spike to their internal integrated state. The CMOS neuron in Figure 5C can be designed containing a current sensing circuit (made of the opamp with resistive feedback) which sets the voltage at the neuron input node *V*_pos_. This current sensing circuit collects all currents provided by all synaptic memristors connected to the neuron input node *V*_pos_. The total instantaneous current sensed drives blocks *V*(*t*) and *C*(*t*) which compute, respectively, the neuron membrane voltage and the calcium variable (see Equation 8). These two instantaneous state variables are monitored by block “*DC level*” which generates a DC output level of three possible values: either zero, positive or negative, according to Equation (9). This DC level is copied to node *V*_pos_ only during times *t*_stdp_ whenever some pre-synaptic neuron starts to spike. If *V*_pos_ = 0 then memristor voltage is equal to *spk*(*t*) during time *t*_stdp_ of a pre-synaptic spike production and is confined within the thresholds in Figure 5D. Under these circumstances, no synaptic strength update is produced. However, if the DC level at *V*_pos_ is set to either the positive or negative output value, the memristor voltage is either shifted up or down during *t*_stdp_, and it will overpass one of the two thresholds, resulting in either an increment or decrement of synaptic strength update. The contribution of one spike to synaptic strength update is controlled by the height of the two first positive and negative steps of *spk*(*t*) during time *t*_stdp_. Both have equal area and thus do not contribute to *V*(*t*) or *C*(*t*) if *V*_pos_ = 0. However, if *V*_pos_ ≠ 0 the symmetry is broken and there would be an undesired contribution. For this reason, current integration at *V*(*t*) and *C*(*t*) needs to be inhibited during times *t*_stdp_. During times *t*_nint_ of *spk*(*t*), the waveform must be such that it always falls below the memristor thresholds and will not affect synaptic weight update. However, its area (shown in green in Figures 5D,E, and which can be made either positive or negative) will contribute to the change in *V*(*t*) and *C*(*t*). This way, the parameters that control synaptic update and shape of function ξ(*t*_pre_) are fully decoupled from the parameters that control neural state variables update. This differs from the case of the conventional double-spike STDP, where these parameters are coupled (Zamarreño-Ramos et al., 2011).

In the case of single-spike STDP, as in the case of the double-spike STDP, if a “*wall model*” memristor is used the resulting STDP learning would be of quadratic type. Otherwise, using a “*filament formation/annihilation*” memristor results in additive STDP learning.

## 6. An application: extracting visual features

Memristors can be used in unsupervised learning models of the visual cortex, and hence extract statistical structure from visual information without requiring supervised labeling. In a first attempt to simulate the early visual system, we used a simple feed-forward set up combining an artificial spiking retina (Lichtsteiner et al., 2008) and a **spiking neural network** mimicking V1 (Zamarreño-Ramos et al., 2011). The artificial retina sensed the external world in a continuous (frame-free) manner, and generated spikes that were asynchronously propagated, as they flowed in, through the feed-forward network. In the V1 layers, neurons were equipped with memristor-based quadratic STDP (simulated). As the system was exposed to natural stimuli, memristors gradually put strong weights on retinal ON- and OFF-center cells with receptive fields aligned in the visual space—because those had correlated spike times—leading to orientation selectivity, in accordance with Hubel and Wiesel's classic model (Hubel and Wiesel, 1959). It is worth mentioning that there was no absolute reference time such as a frame onset, yet information was encoded and decoded in the relative spike times. More recently, we have reproduced these results in a more biologically detailed model, which also included the lateral geniculate nucleus (Masquelier, 2012). Other researchers have followed similar paths with simpler STDP learning functions (as in Figure 4G2) (Bichler et al., 2012b) and proposed PCM-based hardware implementations (Bichler et al., 2012a). Future work will evaluate memristors in subsequent layers, mimicking higher order neurons. We expect that selectivity to more complex visual features will emerge.

Notably, biological hardware is incredibly slow: neurons cannot fire more than a few hundred spikes per second, and those impulses propagate on axons between neurons with a velocity of 1–2 m/s. Spike-driven chips and memristors could be several orders of magnitude faster, and thus could emulate the biological visual system much faster than real time. For example, switching times in the order of nano seconds have been demonstrated for some Hf-based resistive switches of 10×10 nm size (Govoreanu et al., 2011). This is particularly appealing for visual learning, which takes months, if not years, in humans. But there is no reason why memristors could not, for example, extract visual features from huge image databases in a few seconds… We thus speculate that this line of research will yield revolutionary results in the next decade.

## 7. Summary and discussion

In the present paper we reviewed ways of exploiting memristors to implement high density physical neural hardware equipped with STDP. We considered two types of memristor models, the “moving wall” model which results in quadratic STDP, and the “filament formation/annihilation” model which results in a more conventional additive STDP. We also considered two types of STDP rules: the conventional double-spike rule and a more elaborate and biologically realistic single-spike rule. Finally, we briefly reviewed an application for artificial vision learning systems that mimics the operation of the visual cortex.

Large scale neural memristive STDP systems have not been built yet. As memristors are nano-scale devices, they will certainly suffer from significant inter-device parameter mismatch. Querlioz et al. (2011) have analyzed the impact of device parameter mismatch on the performance of STDP learning with memristors using a learning rule similar to the one shown in Figure 4G and have observed very smooth performance degradation even for parameter dispersions as high as 25–50%. Homeostasis, at the neural firing sensitivity level, can be a mechanism to help in compensating synaptic variability (Querlioz et al., 2011). Alternatively, certain STDP functions (like the one in Figure 4G) capable of firing with either one single pre- or post-synaptic spike can induce homeostasis as well (Sjöström and Gerstner, 2010).

In summary, synaptic behavior mismatch is an important concern, but researchers are already proposing possible solutions. It remains, however, to physically build such systems at a large scale and verify *in situ* their reliability, mismatch, and other non-ideal effects, and determine if the proposed solutions are sufficient to make them work reliably and efficiently.

### Conflict of interest statement

The authors declare that the research was conducted in the absence of any commercial or financial relationships that could be construed as a potential conflict of interest.

## Footnotes

^1^Wafer: microchips are fabricated on silicon wafers (with diameter ranging from 1″ to about 18″), which can hold several hundreds or thousands of individual chips that are later on cut and encapsulated into chip packages. The FACETS/BrainScaleS project is a wafer-scale design, meaning that the wafer is not cut into individual chips, but the whole wafer is used as a unit circuit.

^2^ARM stands for “Acorn Risk Microprocessor” and is the name of a company providing embedded microprocessors for a variety of more complex chips, like cellular phones, usb-sticks, etc.

^3^CMOS stands for “Complementary Metal Oxide Semiconductor” and refers to the most standard technology used for microchip fabrication.

^4^In asynchronous systems no clock is required, as opposed to conventional digital computing systems. Consequently, there is no centralized time keeper that enforces actions to happen in lock-step with each other.

^5^By “static” we mean it is not altered by changes of the above electrical quantities, or by their history, integrals, derivatives, etc. These “static” curves can, however, be time-varying if the change is caused by an external agent. For example, a motor driven potentiometer would have a “static” *i*/v curve that is time varying.

## Key Concept

Memristor: Two terminal electronic device which operates similar to a resistor, but whose resistance changes dynamically as the device is being used.
Spike-Timing-Dependent-Plasticity (STDP): One type of learning rule for artificial synapses in spiking neural networks, where the synaptic update depends on the timing characteristics of individual spikes at the synapse terminals.
Artificial learning synapses: Artificially manufactured device that behaves similar to a biological synapse, e.g., it's communication strength (or synaptic weight) changes as the device is used according to some learning rule.
Nanoscale artificial synapse: This is an artificial synapse made using some device whose dimensions are below the micron (10^−6^ m).
Tunable STDP: STDP learning rule whose mathematical description can be made to change in time.
Hybrid nano/CMOS neural system: Artificial neural network system built using conventional microchip technology (CMOS) combined with presently emerging nanoscale devices.
Memristive synapses: Artificial synapses built using memristors.
Spiking Neural Network: Network of neurons that interchange information among them using “spikes.” Spikes are abstractions of biological spikes (also called action potentials). In some electronic spiking neural networks, spikes have similar waveform shapes than in biology, but normally in electronics systems spikes are much simpler being represented by a square digital pulse.
